# The Role of State Public Health Agencies in Addressing Less Prevalent Chronic Conditions

**Published:** 2005-06-15

**Authors:** Fran C Wheeler, Lynda A Anderson, Cynthia Boddie-Willis, Patricia H Price, Mary Kane

**Affiliations:** Association of State and Territorial Chronic Disease Program Directors; Centers for Disease Control and Prevention, Atlanta, Ga, Rollins School of Public Health, Emory University, Atlanta, Ga; Massachusetts Department of Public Health, Boston, Mass; Centers for Disease Control and Prevention, Atlanta Ga; Concept Systems Inc, Ithaca, NY

## Abstract

**Introduction:**

State-based chronic disease programs typically focus on the most prevalent chronic conditions, such as cancer, diabetes, and cardiovascular disease, but interest in less prevalent chronic conditions (LPCCs), such as epilepsy, is growing. In our study, we examined the perceived roles of state health departments in addressing LPCCs and used this information to develop recommendations for state health departments that are considering developing LPCCs programs. We also compared the identified state health department roles for LPCCs with roles related to healthy aging, as well as to the essential elements of existing state-based chronic disease programs, to determine whether future LPCCs programs would have any unique requirements.

**Methods:**

Participants used concept-mapping techniques to generate a set of 100 statements on steps that state health departments could take to address LPCCs. The participants sorted and rated each statement according to importance and feasibility. We used a sequence of multivariate statistical analyses to generate a series of maps, or clusters, and rating graphics. We reviewed the findings and produced recommendations for state health departments. We used a similar process to examine roles of state health departments in addressing healthy aging.

**Results:**

The participants grouped the LPCCs statements into nine clusters, which they rated as moderately feasible and important. The healthy aging statements were grouped into eight clusters. Clusters for LPCCs and healthy aging were similar. We also compared LPCCs clusters and the essential elements of existing state-based chronic disease programs and found that they were similar.

**Conclusion:**

The similarities between LPCCs clusters and essential elements of existing state-based chronic disease programs highlight an important point. State health departments that are considering establishing LPCCs programs should use strategies that have already been used by other public health agencies to develop chronic disease prevention and control programs.

## Introduction

Historically, state-based chronic disease prevention and control programs have focused on the most prevalent chronic conditions, such as cancer, diabetes, and cardiovascular disease. Because these programs have decreased the morbidity and mortality associated with these major conditions, health professionals in various sectors have become more interested in addressing less prevalent chronic conditions (LPCCs) such as epilepsy, Parkinson's disease, multiple sclerosis, and amyotrophic lateral sclerosis (ALS). Unlike prevalent chronic conditions, such as heart disease, that have well-established morbidity and mortality rates in the United States (1), rates for LPCCs are just beginning to be identified. The health ramifications of many LPCCs are as significant as they are for more common chronic diseases; LPCCs simply do not affect as many people. For example, in the United States, cardiovascular disease affects more than 64 million people (2), whereas epilepsy, Parkinson's disease, multiple sclerosis, and ALS combined affect fewer than 4 million people (with epilepsy affecting approximately 2.5 million [3], Parkinson's disease approximately 500,000 [4], multiple sclerosis 250,000 to 350,000 [5], and ALS approximately 20,000 [6]).

Although the role of state-based chronic disease prevention and control programs (referred to as *state health departments*) in addressing more prevalent conditions is gaining attention, their precise roles in and responsibilities for handling LPCCs are not clear. Moreover, state health departments are relatively inexperienced in addressing these conditions. The few health departments with programs for LPCCs such as epilepsy were largely established by legislative directives. Of these, several have small financial initiatives to assist with medical services for low-income patients, and others have used the Behavioral Risk Factor Surveillance System to develop state estimates of epilepsy's prevalence (7,8). These initial efforts lack the requisite public health assessments and planning processes required to address emerging public health issues effectively.

The purpose of our project was to examine the perceptions of people with an interest in public health and LPCCs and determine the perceived feasibility and importance of addressing the conditions through state health departments. We used the data collected to create recommendations for state health departments on ways to address these conditions. Given the long-term partnerships between the Centers for Disease Control and Prevention (CDC) and state health departments, understanding the perceived roles of state health departments is critical. We also hoped to better understand the way state health departments perceive LPCCs relative to other emerging but prevalent public health issues such as healthy aging. Although LPCCs and healthy aging are both complex issues, considered important topics among professionals in the public health arena and other fields, and not characterized as traditional public health issues, aging affects more of the population than all LPCCs combined. In 2002, U.S. Census Bureau estimated that 35.3 million Americans, or 12.6% percent of the population, were aged 65 years or older (9).

In our discussion of state health departments' and stakeholders' perceptions of the roles of state health departments in addressing LPCCs, we compare the roles for LPCCs with the roles for healthy aging, hypothesizing that the roles for both are similar. We then compare the roles for LPCCs with the current roles of state health departments in state-based chronic disease programs (10). This comparison allows us to identify whether state health departments have unique roles in addressing LPCCs and whether these roles require new innovations or more resources than are currently available to state health departments.

The Association of State and Territorial Chronic Disease Program Directors — a nonprofit organization that focuses on chronic disease prevention and control at the state and national levels — began the LPCCs project and the healthy aging project at about the same time, with the LPCCs project beginning in September 2002 and the healthy aging project beginning in August 2002. The projects were conducted independently with support from the CDC.

## Methods

In 1986, Trochim and Linton (11) proposed a general framework to show how conceptualization processes can be used for program planning and evaluation. In 1989, Trochim introduced *concept mapping,* a type of structured conceptualization process that allows the user to identify complex relationships among ideas (12). Concept-mapping techniques were used in both of our projects. As noted by Trochim (12), the concept-mapping method he describes differs from other approaches because it is designed for group use. Participants use focus statements to identify the key elements of a program and represent the relationship of each element to another in the form of an aggregate map (13). Concept mapping has been used for various facets of numerous public health projects, such as setting objectives for use of a tobacco settlement fund and developing an employment program for people with severe mental illness (14-16). The concept-mapping approach is consistent with the participatory methods recommended in the CDC's Framework for Program Evaluation in Public Health (17). Methodological work has been performed to establish the reliability and validity of the concept-mapping methods (12,14)*.*

### LPCCs project

**Procedure. **A project advisory group with members representing state health departments, federal agencies (such as the CDC), organizations that address LPCCs, advocacy groups, and service providers nominated potential participants in the concept-mapping process from groups interested in LPCCs, groups needing LPCCs services, and individuals with LPCCs expertise. The nomination process was part of an effort to elicit a wide range of informed opinions about the role of state health agencies in addressing LPCCs.

We collected data from September 9 through December 18, 2002. The concept mapping took place in two sessions. During the first session, we asked 145 individuals to respond to the following focus statement: "If relatively uncommon chronic conditions (such as epilepsy, multiple sclerosis, and Parkinson's disease) are to be addressed effectively, a specific action, program, or service that state public health agencies should do or facilitate is . . . ." We asked respondents to provide up to 10 brief statements or ideas to the project contractors by e-mail, fax, or regular mail or through the project Web site. We did not collect identifying information from any participant. We sent e-mail reminders twice to improve the response rate. From the 222 ideas generated in the first session, a committee of core project staff members identified 100 applicable, nonrepetitive statements to be used in the second session. Factors such as relevance, redundancy, clarity, and appropriateness were used to produce the final set of 100 statements. (The complete list of statements is available in the final report: www.conceptsystems.com/library/whitepapers.cfm*.)

The second session included a sorting task and a rating task. For the sorting task (18), we asked a subset of 53 participants (with 20 of 53 [38%] actually participating) that were selected for their familiarity with LPCCs to sort the 100 statements into groups, or clusters, based on similarity of ideas. During a 7-week period, participants either sorted the statements into clusters using the project's Web site or manually sorted statements that had been printed on cards. The participants were told the following:

1. Statements cannot be sorted into a single group.
2. Each statement can be placed in only one group.
3. The final number of groups cannot equal the total number of statements.

We informed participants that individuals in similar concept-mapping projects (also with approximately 100 statements) generally created 6 to 15 clusters.

For the rating task in the second session, we asked the original 145 participants to rate the importance and feasibility of each of the 100 statements relative to the other statements. About 50 participants (35% of the 145 invited) assessed importance and feasibility using a 5-point Likert-type response scale ranging from 5 (very important or very feasible) to 1 (not important or not feasible). For each cluster of statements (identified in the first part of the second session), we developed ratings reflecting the sum of scores for each statement in that cluster divided by the total number of statements in the cluster. For the rating task, we collected information on participants' organizational affiliations to compare perceptions among groups. Participants represented 64 different organizations, with the majority representing state health departments (57.8%). Voluntary health agencies (17.2%) and federal health agencies (12.5%) were also represented.

**Data analysis.** An expert in concept mapping used The Concept System software, version 1.75 (Concept Systems Inc, Ithaca, NY) (19) to compile and analyze the sorting and rating data. The software was used to integrate the sorting information and develop a series of maps and reports by applying multidimensional scaling and hierarchical cluster analysis. The analysis involved the following tasks:

1. Constructing a similarity matrix that represented the relative similarity of participants' sorting statements to one another
2. Analyzing the total similarity matrix using nonmetric multidimensional scaling analysis with a two-dimensional solution; generating x and y coordinate locations in two-dimensional space for each statement based on its mathematical similarity to other statements
3. Using a hierarchical cluster analysis to combine the statements into clusters based on the relative x and y coordinates generated by the multidimensional scaling
4. Configuring the multidimensional scaling of the statement points in two dimensions in a point map to serve as a foundation for results development
5. Overlapping the results of the hierarchical cluster analysis on the multidimensional scaling results to create a point cluster map displaying these points graphically within each cluster group, with polygonal boundaries surrounding the points in each cluster group
6. Applying the results of the rating process to the data to produce cluster ratings
7. Conducting a go-zone analysis consisting of a bivariate plot of the average importance and feasibility of each statement cluster

In February 2003, we invited 26 individuals representing members from various organizations and with diverse perspectives for an in-person meeting to review the analysis results and use the results to formulate recommendations for state health departments. Of the 26 invited individuals, 21 attended the meeting. Participants reviewed the cluster analyses, paying particular attention to statements that were rated as highly important and highly feasible. The recommendations they produced for state-based public health programs included specific actions that could be integrated into current state-level activities.

### Healthy aging project

From August 5 to November 25, 2002, we collected data for the healthy aging project using a separate concept-mapping process. We selected representatives from state health departments and stakeholder groups, including state units on aging and various aging-related groups (researchers, policy-making bodies, and community-based organizations). We used the same concept-mapping procedure for the healthy aging project as we used for the LPCCs project. Concept mapping took place in two sessions. During the first session, we asked approximately 248 individuals to respond to the following focus statement: "If new resources were made available to state public health programs to improve the health of older adults, a specific thing that a program should be able to do or provide is . . . ." From the 489 ideas generated in this session, a committee of core project staff members identified 98 applicable, nonrepetitive statements. Factors such as relevance, redundancy, clarity, and appropriateness were used to produce the final set of 100 statements. (The results of the rating tasks for this project are not relevant to this article, but they are available in the final report: www.conceptsystems.com/library/whitepapers.cfm.) During the sorting task, 28 participants (70% of the 40 invited) sorted the statements into clusters. The participants were provided with the same directives as those provided in the LPCCs sorting task. Approximately 107 participants (43% of the 248 invited) then rated the importance and impact of the resulting statements using a 5-point Likert-type response scale. Participants were given approximately 8 weeks to complete the rating task.

### Essential elements of state-based chronic disease programs

We used the CDC report *Promising Practices in Chronic Disease Prevention and Control: A Public Health Framework for Action* (10) to identify seven essential elements of state-based chronic disease programs — elements needed by state health departments to establish comprehensive statewide chronic disease prevention programs. The seven essential elements are as follows: 1) leadership, 2) epidemiology and surveillance, 3) partnerships, 4) comprehensive state plans, 5) interventions, 6) assessment and evaluation, and 7) program management and administration.

## Results

### LPCCs project

**Figure 1:**
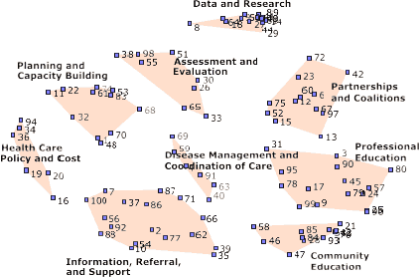
Point-cluster map showing multidimensional scaling arrangement of less prevalent chronic conditions (LPCCs) statements into nine clusters. Each number represents one statement from the first session.

**Map results.** The underlying structure for all maps generated by concept mapping was a point-cluster map that resulted from the arrangements of statements by multidimensional scaling. The participants identified nine clusters that best fit the 100 LPCCs statements (Figure 1) and described ways the results could be used by state health departments. (The list of 100 statements is available from www.conceptsystems.com/library/whitepapers.cfm.) The underlying structure for all maps generated by concept mapping was a point-cluster map that resulted from the arrangements of statements by multidimensional scaling. The participants identified nine clusters that best fit the 100 LPCCs statements (Figure 1) and described ways the results could be used by state health departments. (The list of 100 statements is available from www.conceptsystems.com/library/whitepapers.cfm*.) A core group of respondents reviewed, refined, and selected the following labels for each cluster: 1) assessment and evaluation; 2) community education; 3) data and research; 4) disease management and coordination of care; 5) health care policy and cost; 6) information, referral, and support; 7) partnerships and coalitions; 8) planning and capacity building; and 9) professional education. Note that eight of the clusters surround the disease management and coordination cluster (Figure 1). Its central location indicates that this cluster is linked to all of the other clusters.

**Ratings.** Feasibility ratings for clusters (with 5 being the most feasible and 1 being the least feasible) ranged from 3.32 for partnerships and coalitions (the most feasible) to 2.68 for health care policy and cost (the least feasible). The three clusters rated as having the greatest feasibility were the partnerships and coalitions cluster, the assessment and evaluation cluster, and the professional education cluster. Importance ratings for clusters (with 5 being the most important and 1 being the least important) ranged from 3.77 for health care policy and cost (the most important) to 3.20 for community education (the least important). The three clusters rated as most important were the health care policy and cost cluster, the disease management and coordination of care cluster, and the planning and capacity-building cluster (Table 1).

**Recommendations.** Participants at the February 2003 meeting used the feasibility and importance cluster ratings, in addition to bivariate plots of the average importance and feasibility of each statement in a cluster, to create the following five recommendations for state health departments:

1. Frame the problem to be addressed, and document the burden associated with LPCCs.
2. Establish strong working relationships with other government agencies and nongovernmental lay and professional groups.
3. Use data and work with partners to develop comprehensive state plans to guide program efforts.
4. Identify priorities for change (e.g., populations, organizations, environments), choose the best channels through which to reach the identified targets, and select appropriate strategies for change.
5. Use systematic approaches to determine whether programs to address LPCCs are being implemented successfully and objectives are being met.

### Healthy aging clusters compared with LPCCs clusters

**Figure 2:**
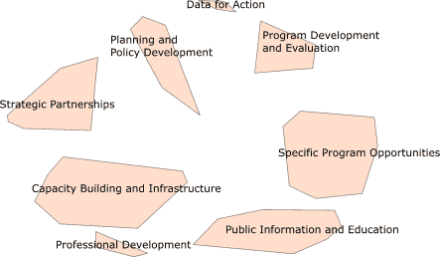
Cluster map showing multidimensional scaling arrangement of healthy aging statements into eight clusters.

In the healthy aging project, participants grouped the 98 statements into eight clusters (Figure 2): 1) capacity building and infrastructure, 2) data for action, 3) planning and policy development, 4) professional development, 5) program development and evaluation, 6) public information and education, 7) specific program opportunities, and 8) strategic partnerships*.* Because the final clusters for LPCCs and healthy aging were similar (Table 2), we only compared the LPCCs clusters (not the healthy aging clusters) with the essential elements of state-based chronic disease programs (10).

### Comparison of LPCCs clusters and essential elements of state-based chronic disease programs

**Figure 3:**
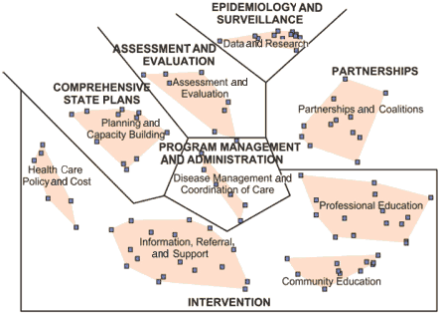
Final concept map for less prevalent chronic conditions (LPCCs) showing clusters and their relationships to clusters from the Centers for Disease Control and Prevention (CDC) report *Promising Practices in Chronic Disease Prevention and Control* (10).

We superimposed the cluster map for LPCCs onto the essential elements of state-based chronic disease programs identified in the CDC's *Promising Practices in Chronic Disease Prevention and Control* (10) (Figure 3). The four clusters at the top of the map in Figure 3 align with four essential elements of chronic disease programs: planning and capacity building aligns with comprehensive state plans; assessment and evaluation aligns with assessment and evaluation; data and research aligns with epidemiology and surveillance; and partnerships and coalitions aligns with partnerships. In addition, the lower four project clusters (health care policy and cost; information, referral, and support; community education; and professional education) fall within the intervention program element. The central cluster, disease management and coordination of care, aligns most closely with the essential service of program management and administration. The central location of this cluster suggests that it may function as a strategic link to the other clusters. Indeed, the final priority recommendations for state health departments are similar to several of the individual statements or ideas in the disease management and coordination of care cluster, such as the recommendations to create comprehensive chronic disease programs and identify and promote best practices. The one essential element of state-based chronic disease programs missing from the LPCCs clusters is leadership.

## Discussion

Recommendations for state health departments interested in LPCCs tended to focus on feasibility rather than importance. Of the nine clusters that emerged, the three clusters considered the most feasible were 1) partnerships and coalitions, 2) assessment and evaluation, and 3) professional education. Half of the recommendations focused on partnership issues, the cluster rated the most feasible. This included the recommendation to establish strong working relationships with other groups and the recommendation to work with partners to develop comprehensive state plans to guide program efforts.

The findings have been disseminated to all project participants, the directors of all state chronic disease programs, and participants in Living Well With Epilepsy II: A Conference on Current Issues and Future Strategies, the second national conference on public health and epilepsy, which was held in July 2003. Anecdotal feedback suggests that the project results were useful in establishing a framework for state-level discussions about developing programs to address LPCCs.

As expected, the clusters for LPCCs were similar to those identified in the healthy aging project. Overall, these two projects and their resultant conceptualization of the roles for public health agencies in addressing LPCCs underscored the fact that the participant groups, which included many who had previously had little or no contact with state health departments, viewed the roles of state health departments similarly despite differences in the issues' prevalence.

With the notable exception of leadership, LPCCs clusters reflected the roles of state health departments identified in the essential elements of state-based chronic disease programs (10). Project discussions about leadership and the absence of a leadership cluster seem to indicate that public health agencies are reluctant to take the lead in initiating new programs such as those addressing LPCCs. State health departments have had little or no involvement in addressing LPCCs. They have other competing priorities such as cardiovascular health, a more prevalent condition. In addition, because this project occurred about 1 year after the events of September 11, 2001, state-based bioterrorism preparedness activities were also demanding attention. LPCCs were rated as only moderately important. As a result, partners of state health agencies need to ensure that any recommendations on LPCCs can be easily incorporated into ongoing activities to be mutually beneficial.

Several factors should be considered when reviewing these findings. The study results should not be interpreted as representing the views of all who work on behalf of people with LPCCs. Participants were primarily individuals directly involved in state-level funding and policy implementation for LPCCs. Although we attempted to include participants who were directly affiliated with disease-specific organizations, given the focus on state health departments, almost 90% of the participants were members of organizations with a more general health care focus.

We used a single focus question to determine the role of states in addressing LPCCs. The question did not specify a particular medical condition, form of response, or responsibility. Additional work focusing on a single medical condition, such as epilepsy or multiple sclerosis, could generate data that are more specific for that condition. Lack of specificity in this project resulted in general recommendations, such as identifying priorities and channels for change, that require further refinement and elaboration by public health programs that have an interest in LPCCs. For example, state health departments' readiness to address one or more of the identified recommendations needs to be determined.

The dynamics of participating in a study asynchronously using the Internet is qualitatively different from completing a typical (i.e., paper or e-mail) survey, so it is possible that this affected participation rates and the content of submitted ideas. However, participants reported that the Internet-based system was easy to use, particularly for the initial session. People who were uncomfortable with the computer interface had the option of submitting suggestions by mail or fax.

We found that concept mapping was an effective approach for engaging state health department representatives and a diverse group of stakeholders nationwide. The project's Internet-based design allowed stakeholders in numerous geographical locations to participate online rather than in person. The collaborative concept-mapping process resulted in collective input into the ideas, clusters, and recommendations that resulted from the project. The concept-mapping technique resulted in a set of recommendations for state health departments to consider when addressing LPCCs. Finally, the importance ratings can be used to monitor progress and can be revisited in the future.

The identified roles of state health departments in addressing LPCCs mirror the essential elements of public health programs for chronic disease prevention and control. These findings reinforce how important it is for state health departments that are considering developing LPCCs programs to use strategies already in place by public health agencies with chronic disease prevention and control programs.

## Figures and Tables

**Table 1:** Feasibility and Importance Ratings for Less Prevalent Chronic Conditions (LPCCs) Clusters

**Cluster Name**	**Feasibility**		**Importance**	
	**Rating^a^**	**Rank**	**Rating^b^**	**Rank**
Partnerships and coalitions	3.32	1	3.54	5
Assessment and evaluation	3.21	2	3.55	4
Professional education	3.10	3	3.34	7
Planning and capacity building	3.02	4	3.59	3
Disease management and coordination of care	2.97	5	3.61	2
Community education	2.86	6	3.20	9
Data and research	2.80	7	3.49	6
Information, referral, and support	2.78	8	3.27	8
Health care policy and cost	2.68	9	3.77	1

a Scale of 1-5; higher scores reflect greater feasibility.

b Scale of 1-5; higher scores reflect greater importance.

**Table 2:** Comparison of Clusters for Healthy Aging and Less Prevalent Chronic Conditions (LPCCs)

**Healthy Aging Clusters**	**LPCCs Clusters**
Strategic partnerships	Partnerships and coalitions
Program development and evaluation	Assessment and evaluation
Data for action	Data and research
Planning and policy development	Planning and capacity building
	Health care policy and cost
Capacity building and infrastructure	Planning and capacity building
Specific program opportunities	Disease management and coordination of care
Professional development	Professional education
Public information and education	Information, referral, and support
	Community education

## References

[1] Mackay J, Mensah GA The atlas of heart disease and stroke, 2004.

[2] American Heart Association (2003). Heart disease and stroke statistics — 2004 update.

[3] Epilepsy Foundation [homepage on the Internet].

[4] National Institute of Neurological Disorders and Stroke Parkinson's disease: hope through research.

[5] National Institute of Neurological Disorders and Stroke Multiple sclerosis: hope through research.

[6] National Institute of Neurological Disorders and Stroke NINDS amyotrophic lateral sclerosis information page.

[7] Centers for Disease Control and Prevention (2001). Health-related quality of life among persons with epilepsy — Texas, 1998. MMWR Morb Mortal Wkly Rep.

[8] Kobau R, DiIorio CA, Price PH, Thurman DJ, Martin LM, Ridings T (2004). Prevalence of epilepsy and health status of adults with epilepsy in Georgia and Tennessee: Behavioral Risk Factor Surveillance System, 2002. Epilepsy Behav.

[9] U.S. Census Bureau International database. Table 094. Midyear population, by age and sex [Internet].

[10] Centers for Disease Control and Prevention Promising practices in chronic disease prevention and control: a public health framework for action [Internet].

[11] Trochim W, Linton R (1986). Conceptualization for evaluation and planning. Eval Program Plann.

[12] Trochim W (1989). An introduction to concept mapping for planning and evaluation. Eval Program Plann.

[13] Shern DL, Trochim WM, LaComb CA (1995). The use of concept mapping for assessing fidelity of model transfer: an example from psychiatric rehabilitation. Eval Program Plann.

[14] Trochim WM (1989). Concept mapping: soft science or hard art?. Eval Program Plann.

[15] Trochim WM, Milstein B, Wood BJ, Jackson S, Pressler V (2004). Setting objectives for community and systems change: an application of concept mapping for planning a statewide health improvement initiative. Health Promot Pract.

[16] Trochim WM, Cook JA, Setze R (1994). Using concept mapping to develop a conceptual framework of staff's views of a supported employment program for persons with severe mental illness. J Consult Clin Psychol.

[17] Centers for Disease Control and Prevention (1999). Framework for program evaluation in public health. MMWR Recomm Rep.

[18] Rosenberg S, Kim MP (1975). The method of sorting as a data gathering procedure in multivariate research. Multivariate Behav Res.

[19] Concept Systems Incorporated The Concept System Version 1.75 software.

